# Cardiovascular Complications of COVID-19 in Athletes: A Systematic Review and Meta-analysis

**DOI:** 10.7759/cureus.87675

**Published:** 2025-07-10

**Authors:** Ali M Zahyan, Hafsah H Alhakami, Abdullah H Khormi, Nawaf S Alhufayyan, Mohammed A AlQarni, Abdulaziz M Alrashidi

**Affiliations:** 1 Cardiology, Prince Mohammed Bin Abdulaziz Hospital, Riyadh, SAU; 2 Internal Medicine, King Fahad Central Hospital, Jazan, SAU; 3 Internal Medicine, Armed Forces Hospital, Aseer, SAU

**Keywords:** athletes, athletic heart, cardiovascular complications, covid-19, myocarditis, myopericarditis, pericardial effusion, pericarditis, sars-cov-2, sports cardiology

## Abstract

This systematic review and meta-analysis aimed to assess the prevalence of cardiovascular complications associated with coronavirus disease 2019 (COVID-19) infection in athletes. A comprehensive search was conducted across PubMed, Web of Science, Scopus, and the Virtual Health Library using the terms (“COVID-19” OR “SARS-CoV-2”) AND (“athletes” OR “athlete”) AND (“pericarditis” OR “myocarditis” OR “pericardial effusion” OR “cardiovascular” OR “cardiac”). Of 671 records, 20 studies met the inclusion criteria. The most commonly reported cardiovascular abnormality was pericardial effusion, with a pooled prevalence of 1.9% (95% CI 0.08-4.4), followed by myocarditis (1.5%; 95% CI 0.9-2.7), pericarditis (1.3%; 95% CI 0.8-2.1), and myopericarditis (0.9%; 95% CI 0.2-3.4). No cases of cardiovascular or all-cause mortality were reported among athletes with COVID-19. These findings suggest that cardiovascular complications are rare in athletic populations following COVID-19 infection, potentially reflecting the protective effect of a robust immune system and high baseline cardiovascular fitness.

## Introduction and background

The outbreak of coronavirus disease 2019 (COVID-19) was first reported in late 2019 in Wuhan, China, and rapidly spread across continents. By early 2020, the World Health Organization (WHO) declared it a global public health emergency. Since then, the pandemic has virtually affected every country, with over 680 million confirmed cases and nearly 7 million deaths worldwide [1]. COVID-19 exhibits a wide clinical spectrum, ranging from mild symptoms such as fever, cough, fatigue, and anosmia to severe respiratory distress requiring mechanical ventilation and intensive care unit admission [2].

Beyond its respiratory manifestations, COVID-19 has been increasingly associated with cardiovascular complications, including myocardial injury, myocarditis, acute coronary syndrome, thromboembolism, cardiomyopathy, and arrhythmias [3], which can significantly affect morbidity and mortality. The risk of cardiovascular involvement is notably higher among older adults, individuals with underlying comorbidities, and those experiencing severe forms of the disease [3]. Several mechanisms have been proposed to explain this association, including cytokine-mediated inflammation, endothelial dysfunction, coagulopathy, direct viral invasion of cardiac tissue, and adverse drug effects [4].

Regular physical activity has been recognized as a protective factor against both the incidence and severity of COVID-19 infection [5]. Athletes, who generally represent a younger and healthier subset of the population, tend to experience asymptomatic or mild infections. Nevertheless, emerging evidence indicates that even this population is not immune to cardiac complications. Cases of myocarditis, pericarditis, and pericardial effusion have been documented in athletes following SARS-CoV-2 infection [6,7]. For instance, a study by Maestrini et al. identified four athletes with pericardial effusion and two with myocarditis following COVID-19 infection [8]. Similarly, Bhatia et al. reported nine cases of pericarditis and eight of myocarditis in affected athletes [9].

Given the potential for cardiovascular involvement even in low-risk, highly active individuals, it is important to understand the prevalence and nature of these complications in athletes. In this systematic review and meta-analysis, we aimed to assess the prevalence of cardiovascular complications among athletes infected with COVID-19 and to provide a synthesis of the available evidence.

## Review

Methods

Search Strategy

**Figure 1:**
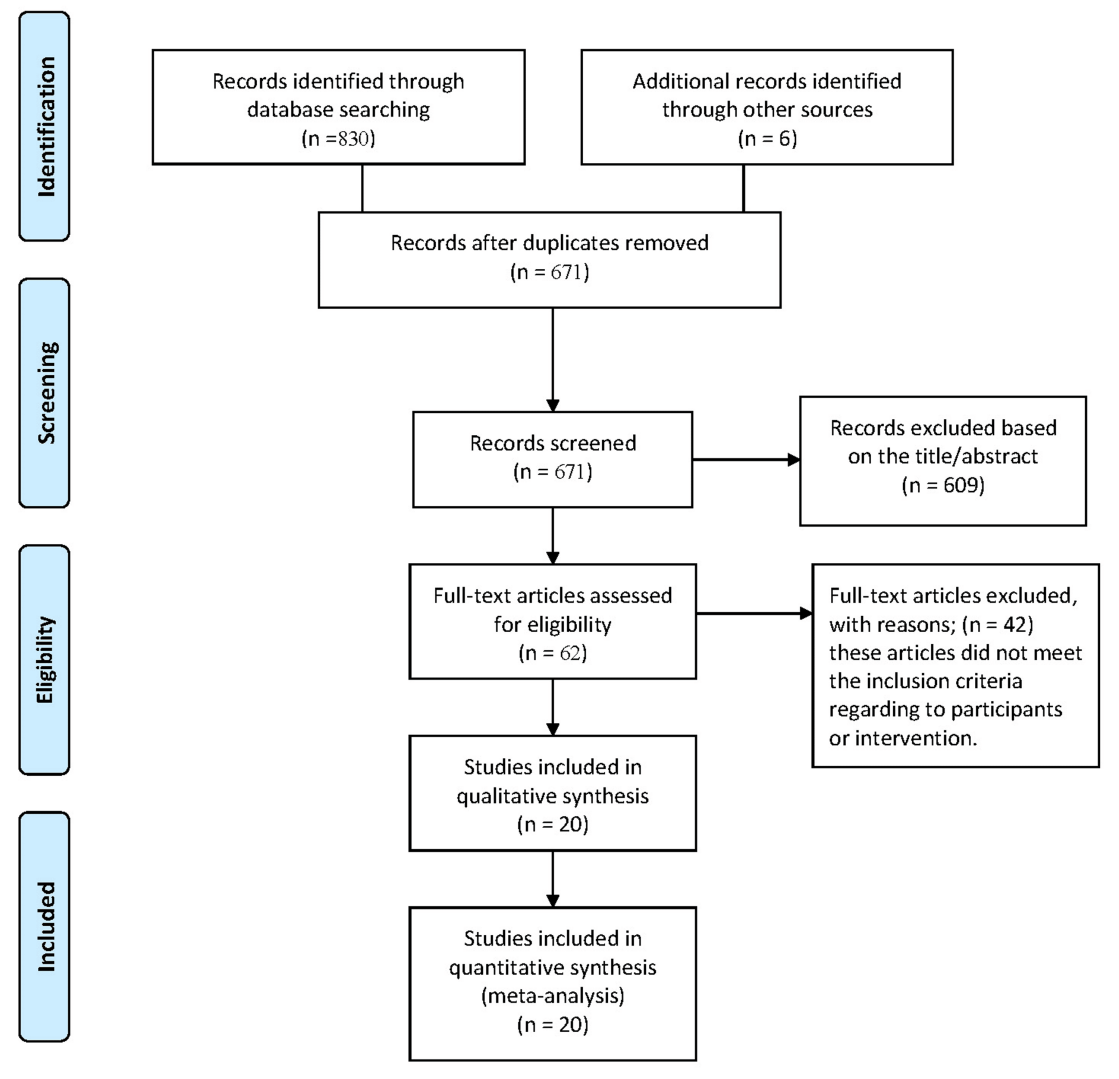
PRISMA flow diagram depicting the study selection process

This systematic review and meta-analysis was conducted according to the Preferred Reporting Items for Systematic Reviews and Meta-Analyses (PRISMA) guidelines [10]. A comprehensive literature search was conducted on April 23, 2023, across four electronic databases: PubMed, Scopus, Web of Science, and the Virtual Health Library. The search strategy used the terms: (“COVID-19” OR “SARS-CoV-2”) AND (“athletes” OR “athlete”) AND (“pericarditis” OR “myocarditis” OR “pericardial effusion” OR “cardiovascular” OR “cardiac”). Full search results from each database are summarized in Figure 1.

Eligibility Criteria

Studies that reported cardiovascular complications in athletes infected with COVID-19, specifically myocarditis, pericarditis, pericardial effusion, or other cardiac pathologies were included. Eligible study designs were cohort studies, cross-sectional studies, and case series. We excluded review articles, case reports, books, conference abstracts, and studies lacking full-text availability or relevant outcome data.

Study Selection and Screening

All search results were imported into EndNote (Clarivate, London, UK) and then transferred to Microsoft Excel (Microsoft Corp., Redmond, WA, USA) for screening and evaluation. Title and abstract screening were followed by full-text review, conducted independently by at least three authors. Disagreements were resolved through open discussion and a consensus-based approach.

Data Extraction and Quality Assessment

A standardized data extraction form was developed by one author with input from the senior author. The form captured key study characteristics (author, publication year, country, study design, sample size, participant age, and percentage of male subjects) and outcome data (prevalence of cardiovascular complications). Data were extracted by three or more authors and subsequently reviewed by another independent author. Risk of bias was assessed using the National Institutes of Health quality assessment tool for observational cohort and cross-sectional studies [11]. Each study was rated as “good,” “fair,” or “poor.” 

Statistical Analysis

All analyses were performed using Comprehensive Meta-Analysis software (version 3.0). We calculated pooled prevalence estimates with 95% confidence intervals for each cardiovascular outcome. Statistical heterogeneity was assessed using the chi-square test and the I² statistic. A random-effects model was applied when heterogeneity was significant (p < 0.05); otherwise, a fixed-effects model was used. For outcomes reported in 10 or more studies, publication bias was evaluated using Egger’s test [12-14].

Results

Study Selection

A total of 671 records were identified through database searches. Following title and abstract screening, 62 full-text articles were reviewed to determine eligibility. Of these, 14 studies met the inclusion criteria. An additional six studies were identified through manual search methods, resulting in a total of 20 studies included in the final meta-analysis. A summary of the quality assessment of the included studies is presented in Table 1.

**Table 1:** Quality assessment of cross-sectional and cohort studies included in the review The quality of each study was assessed using the National Institutes of Health quality assessment tool for observational cohort and cross-sectional studies [11]. The total score represents the number of criteria met by each study. Studies were rated as good (12–14 points), fair (7–11 points), or poor (0–6 points). The year and country of the study are provided alongside the reference number for clarity. Reference numbers correspond to the numbered list of citations included in the manuscript.

Study ID	Year	Country	Total score	Rating
Daniels et al. [6]	2021	USA	11	Fair
Erickson et al. [7]	2021	USA	11	Fair
Maestrini et al. [8]	2023	Italy	10	Fair
Bhatia et al. [9]	2023	Multicenter	11	Fair
Bavishi et al. [15]	2023	USA	12	Good
Brito et al. [16]	2021	USA	10	Fair
Cavigli et al. [17]	2021	Italy	10	Fair
Clark et al. [18]	2021	USA	11	Fair
Colangelo et al. [19]	2022	Italy	11	Fair
Hendrickson et al. [20]	2021	USA	11	Fair
Krzywański et al. [21]	2022	Poland	10	Fair
Mascia et al. [22]	2021	Italy	11	Fair
Małek et al. [23]	2020	Poland	10	Fair
Martinez et al. [24]	2021	USA	10	Fair
Moulson et al. [25]	2021	USA	11	Fair
Mitrani et al. [26]	2022	USA	11	Fair
Rasmusen et al. [27]	2022	Denmark	11	Fair
Starekova et al. [28]	2021	USA	11	Fair
Szabó et al. [29]	2022	Hungary	11	Fair
Rajpal et al. [30]	2021	USA	10	Fair

Study Characteristics

The 20 included studies consisted of 7 retrospective cohort studies, 7 cross-sectional studies, and 6 prospective cohort studies. Collectively, these studies involved 11,182 athletes diagnosed with COVID-19. Sample sizes varied widely, ranging from 26 to 3,653 participants. Eleven studies were conducted in the USA, followed by four in Italy, two in Poland, and one study each from Denmark and Hungary. One additional study had a multicenter design involving multiple countries. The demographic and clinical characteristics of the study populations are summarized in Table 2. 

**Table 2:** Characteristics of the included studies Median age is denoted with an asterisk.

Study ID	Year	Country	Study design	Sample size	Age (mean or median, years)	Male (%)
Daniels et al. [6]	2021	USA	Retrospective cohort	1597	-	-
Erickson et al. [7]	2021	USA	Retrospective cohort	170	19.5	54
Maestrini et al. [8]	2023	Italy	Cross-sectional	219	23*	59
Bhatia et al. [9]	2022	Multicenter	Retrospective cohort	511	21*	88
Bavishi et al. [15]	2023	USA	Retrospective cohort	3653	19.9	67
Brito et al. [16]	2020	USA	Cross-sectional	54	19*	85
Cavigli et al. [17]	2021	Italy	Cross-sectional	90	24	71
Clark et al. [18]	2021	USA	Retrospective cohort	59	20*	37
Colangelo et al. [19]	2022	Italy	Retrospective cohort	77	18	79
Hendrickson et al. [20]	2021	USA	Retrospective cohort	137	20*	68
Krzywański et al. [21]	2022	Poland	Cross-sectional	111	22	47
Mascia et al. [22]	2021	Italy	Prospective cohort	58	-	-
Małek et al. [23]	2020	Poland	Cross-sectional	26	24*	19
Martinez et al. [24]	2021	USA	Cross-sectional	789	25	98.5
Moulson et al. [25]	2021	USA	Prospective cohort	3018	20	-
Mitrani et al. [26]	2022	USA	Prospective cohort	174	21*	70
Rasmusen et al. [27]	2022	Denmark	Prospective cohort	121	24.7	62
Starekova et al. [28]	2021	USA	Retrospective cohort	145	19.6	75
Szabó et al. [29]	2022	Hungary	Prospective cohort	147	23	64
Rajpal et al. [30]	2021	USA	Cross-sectional	26	19.5	58

Prevalence of Cardiovascular Complications

**Figure 2:**
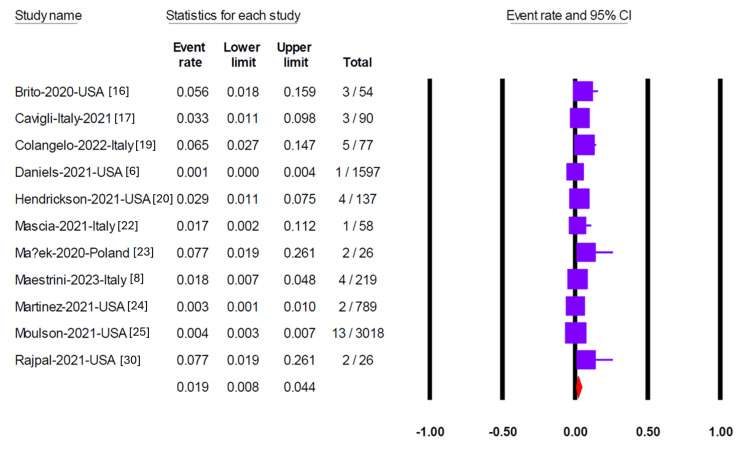
Forest plot showing the pooled prevalence of pericardial effusion in included studies Forest plot illustrating the pooled prevalence of pericardial effusion across 11 studies, with an overall prevalence of 1.9% (95% CI 0.08–4.4). Egger’s test showed no significant publication bias (p = 0.35).

**Figure 3:**
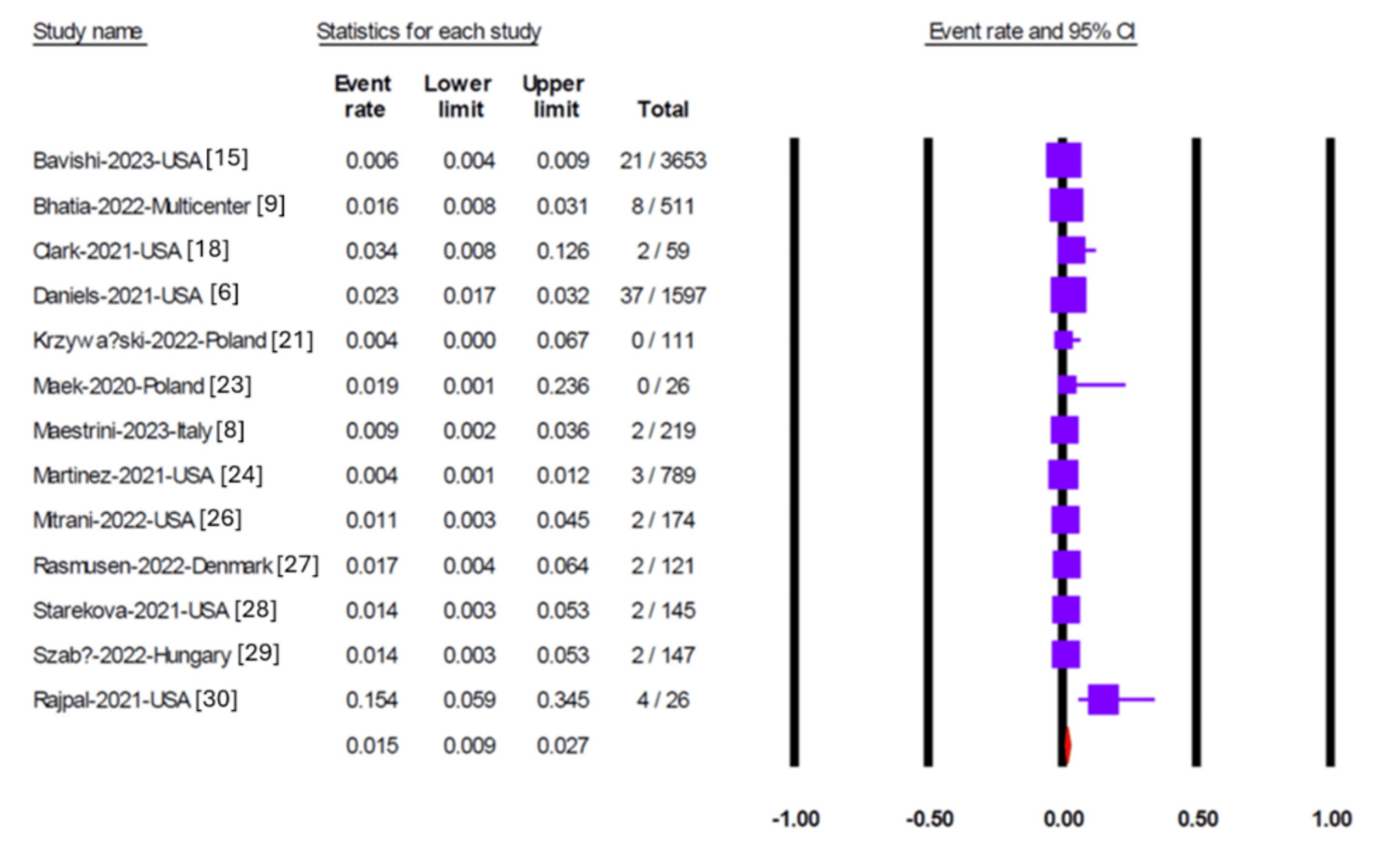
Forest plot showing the pooled prevalence of myocarditis in included studies Forest plot illustrating the pooled prevalence of myocarditis from 13 studies, including two studies reporting no cases, with an overall pooled prevalence of 1.5% (95% CI 0.9–2.7). Egger’s test indicated no evidence of publication bias (p = 0.95).

**Figure 4:**
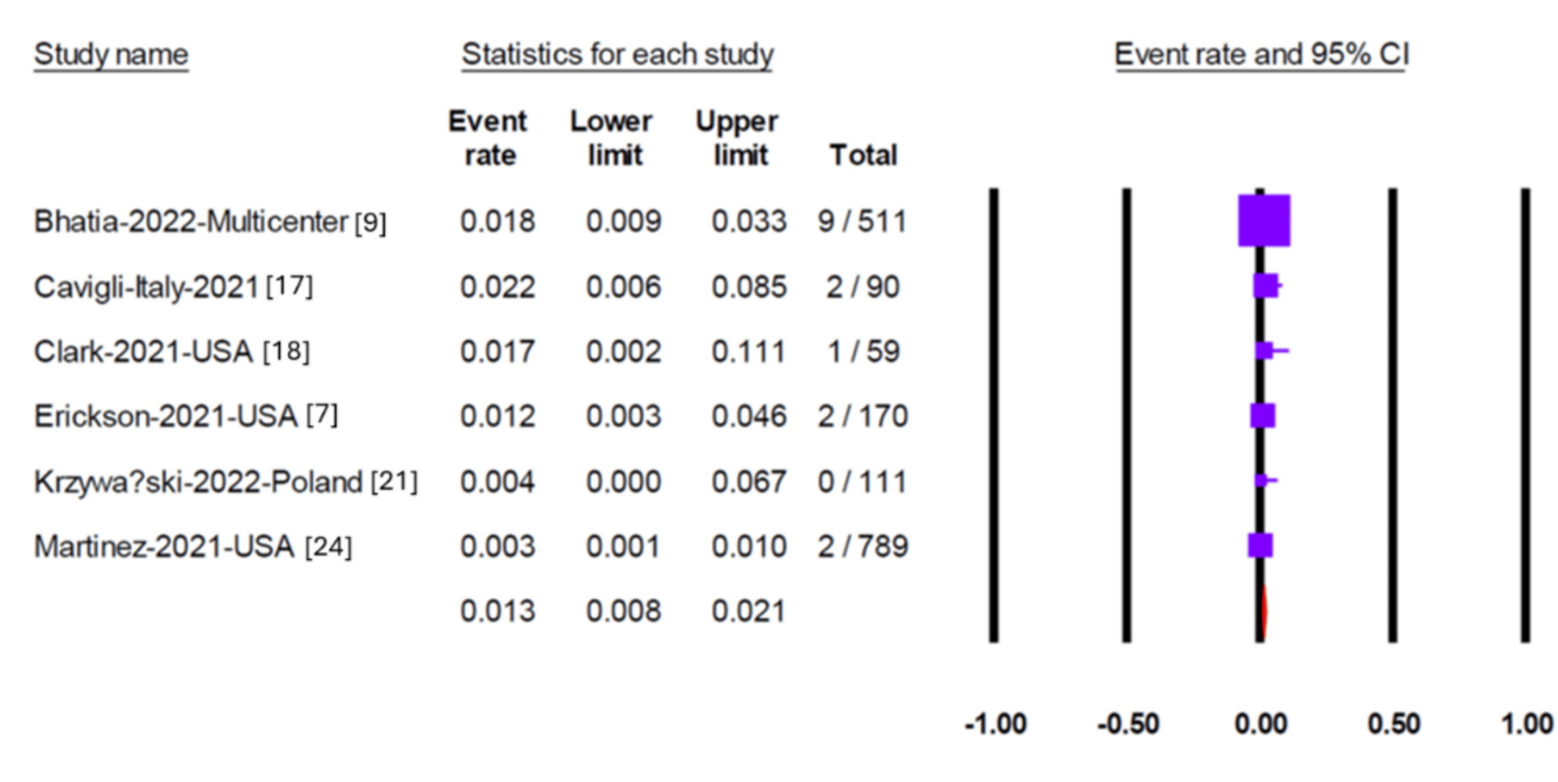
Forest plot showing the pooled prevalence of pericarditis in included studies Forest plot showing the pooled prevalence of pericarditis from six studies, with an overall prevalence of 1.3% (95% CI 0.8–2.1).

**Figure 5:**
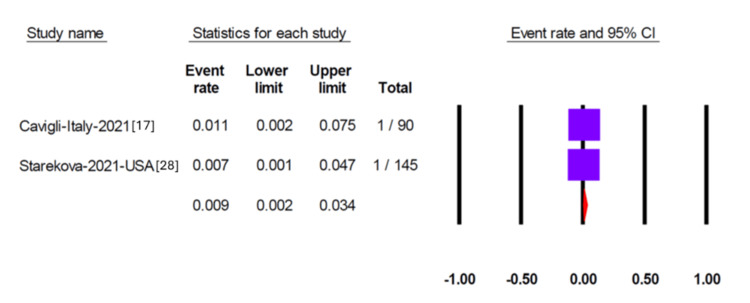
Forest plot showing the pooled prevalence of myopericarditis in included studies Forest plot depicting the pooled prevalence of myopericarditis reported in two studies, showing a prevalence of 0.9% (95% CI, 0.2–3.4).

Pericardial effusion was reported in 11 studies, yielding a pooled prevalence of 1.9% (95% CI 0.08-4.4) (Figure 2). Egger’s test did not detect significant publication bias (p = 0.35). Myocarditis was assessed in 13 studies, two of which reported no cases. The overall pooled prevalence of myocarditis was 1.5% (95% CI 0.9-2.7) (Figure 3), with no evidence of publication bias (Egger’s test, p = 0.95). Six studies reported on pericarditis, with a pooled prevalence of 1.3% (95% CI 0.8-2.1) (Figure 4). Myopericarditis was less commonly reported, with only two studies addressing this outcome; the pooled prevalence was 0.9% (95% CI 0.2-3.4) (Figure 5).

Mortality and Other Cardiovascular Events

Notably, none of the included studies reported any cardiovascular or all-cause mortality among athletes with confirmed COVID-19 infection. Additionally, other major cardiovascular events were rarely reported. One study documented two cases of coronary artery ectasia among 137 athletes [20], while another large-scale study involving 3653 participants reported no instances of myocardial infarction or pulmonary embolism [15].

Discussion

This meta-analysis assessed the prevalence of cardiovascular complications among athletes infected with COVID-19 and found that such complications are relatively rare. Among the included studies, pericardial effusion had the highest pooled prevalence at 1.9%, followed by myocarditis (1.5%), pericarditis (1.3%), and myopericarditis (0.9%). These findings are consistent with previously reported estimates and suggest that most athletes, who typically have mild or asymptomatic infections, are not at high risk for serious cardiac events [6,7,9,15].

Myocarditis remains a particular concern due to its association with sudden cardiac death in athletes. Although the overall prevalence was low, it is clinically significant. The consistency of prevalence across studies, including those by Daniels et al. [6], Erickson et al. [7], and Bhatia et al. [9], suggests a reproducible risk profile in this population. Some studies, such as Krzywański et al. [21], reported no cases of myocarditis, likely due to differences in screening protocols, imaging modalities, or population characteristics.

Pericardial effusion, the most frequently observed abnormality, may often be subclinical. While it was more common than other findings, its clinical significance varies. In many athletes, especially those undergoing detailed cardiac MRI screening, small or incidental effusions may be detected without any symptoms [8,16]. The variation in reported prevalence is likely due to differing definitions, diagnostic tools, and thresholds for abnormal findings.

Pericarditis and myopericarditis were reported less frequently, but their presence highlights the spectrum of inflammatory responses in the post-infectious phase. The similar prevalence rates of pericarditis and myocarditis in some studies reflect their overlapping pathophysiological mechanisms [9,17,18].

Crucially, no cardiovascular or all-cause mortality was reported across all 20 included studies. This finding is consistent with the recognized lower risk of severe COVID-19 outcomes in younger, healthier individuals with high baseline cardiorespiratory fitness [15,16,24]. Athletes may benefit from more effective immune regulation and lower systemic inflammation, potentially offering protection against severe cardiac involvement [5,24].

Despite the low rates of clinical complications, concerns remain regarding subclinical cardiac changes. Several studies using cardiac magnetic resonance imaging identified myocardial edema or late gadolinium enhancement in athletes without symptoms [6,22,23]. The long-term implications of these findings are not yet fully understood, but they reinforce the need for careful evaluation before resumption of high-intensity physical activity.

Limitations

This study has several limitations. First, there was notable heterogeneity among the included studies, likely due to differences in study design, diagnostic criteria, imaging protocols, and population characteristics. Second, many studies were cross-sectional, limiting the ability to assess causality or long-term outcomes. Third, most studies did not specify the type or intensity of sport, preventing subgroup analysis by athletic discipline. Finally, the reliance on published data may introduce publication bias, particularly for rare outcomes or studies with negative findings.

## Conclusions

Cardiovascular complications associated with COVID-19 infection are infrequent among athletes, with pericardial effusion, myocarditis, and pericarditis occurring at low prevalence rates and no mortality reported across included studies. This suggests that factors such as younger age, high levels of physical fitness, and a well-regulated immune system may provide a protective effect against severe cardiac involvement in this population. Nonetheless, given the potential for subclinical cardiovascular abnormalities and the serious implications of myocarditis, careful screening and follow-up remain essential to ensure athlete safety and guide return-to-play decisions. Future research should focus on long-term cardiovascular outcomes and the impact of different sports disciplines.
